# Fabrication of Phosphorus-Doped Cobalt Silicate with Improved Electrochemical Properties

**DOI:** 10.3390/molecules26206240

**Published:** 2021-10-15

**Authors:** Jie Ji, Yunfeng Zhao, Yifu Zhang, Xueying Dong, Changgong Meng, Xiaoyang Liu

**Affiliations:** 1 State Key Laboratory of Inorganic Synthesis and Preparative Chemistry, College of Chemistry, Jilin University, Changchun 130012, China; jijie1218@mails.jlu.edu.cn; 2 State Key Laboratory of Fine Chemicals, School of Chemical Engineering, Dalian University of Technology, Dalian 116024, China; yfdlut@163.com (Y.Z.); yfzhang@dlut.edu.cn (Y.Z.); dxy1123@mail.dlut.edu.cn (X.D.); cgmeng@dlut.edu.cn (C.M.)

**Keywords:** Co_2_SiO_4_, phosphorus-doped, supercapacitor electrode, electrochemical performances

## Abstract

The development of electrode materials for supercapacitors (SCs) is greatly desired, and this still poses an immense challenge for researchers. Cobalt silicate (Co_2_SiO_4_, denoted as CoSi) with a high theoretical capacity is deemed to be one of the sustainable electrode materials for SCs. However, its achieved electrochemical properties are still not satisfying. Herein, the phosphorus (P)-doped cobalt silicate, denoted as PCoSi, is synthesized by a calcining strategy. The PCoSi exhibits 1D nanobelts with a specific surface area of 46 m^2^∙g^−1^, and it can significantly improve the electrochemical properties of CoSi. As a supercapacitor’s (SC’s) electrode, the specific capacitance of PCoSi attains 434 F∙g^−1^ at 0.5 A∙g^−1^, which is much higher than the value of CoSi (244 F∙g^−1^ at 0.5 A∙g^−1^). The synergy between the composition and structure endows PCoSi with attractive electrochemical properties. This work provides a novel strategy to improve the electrochemical performances of transition metal silicates.

## 1. Introduction

In the past decades, supercapacitors (SCs) have been greatly focused on owing to their environmentally friendly, long cycle life, high-power density and comparatively low cost [1,2,3,4]. The design and development of electrode materials with sustainable sources and excellent performances are great challenges and essential for the large-scale application of SCs [5,6,7,8]. In terms of achieving high-performance SCs, the electrode materials must possess a high safety, large theoretical capacitance, long cycle life as well as low cost [9,10,11,12,13,14].

Transition metal silicates (TMSs) possess the above features of high-performance SCs [15,16,17,18,19,20,21,22,23]. Among TMSs, Cobalt-based silicates as electrode materials have received great interest because they can be used as promising electrode materials for batteries, SCs and water splitting owing to their abundance, low cost and stability [24,25,26,27]. Due to the desirable theoretical capacity of cobalt silicate (Co_2_SiO_4_, denoted as CoSi), a lot of attention has been paid to CoSi-related materials [13,28,29]. However, so far, the electrochemical properties of CoSi for SCs are far from being sufficient owing to its poor electroconductivity [30,31,32]. Thus, many studies have focused on the design and preparation of CoSi-based materials to improve the electrochemical properties of CoSi in recent years [33,34,35,36,37,38,39]. For example, Dong et al. synthesized the sandwich-like CoSi/reduced graphene oxide (rGO)/CoSi (CoSi/rGO/CoSi) architecture using SiO_2_/rGO/SiO_2_ as the template by the hydrothermal method. This showed enhanced electrochemical performances of ≈429 F·g^−1^ at 0.5 A·g^−1^ and ≈92% after 10,000 cycles [36]. Cheng et al. synthesized the coupled CoSi nanobelts@CoSi with rGO, and this exhibited a remarkable capacitive performance with ≈483 F g^−1^ at 0.5 A g^−1^ [30]. Until now, several strategies, including coating [37,38], composites with graphene [35], biomass technique [33], novel structures [13], etc. have been adopted to synthesize CoSi-based architectures with improved electrochemical properties of CoSi. Despite the above advances, the electrochemical performances of CoSi-based materials for SCs still need to be further studied. Therefore, it is greatly challenging for researchers to develop new strategies to construct CoSi-based materials with enhanced electrochemical properties.

In this work, phosphorus (P) is introduced to incorporate CoSi (denoted as PCoSi) by a calcining route, and the electrochemical properties of CoSi are greatly enhanced. The strong nucleophilic P will introduce much more reactive sites for CoSi, which is conducive to the electrochemical reaction’s efficiency. This work offers a calcining strategy to synthesize PCoSi, which can be considered as a promising candidate for the SCs’ material.

## 2. Results and Discussion

**Figure 1:**
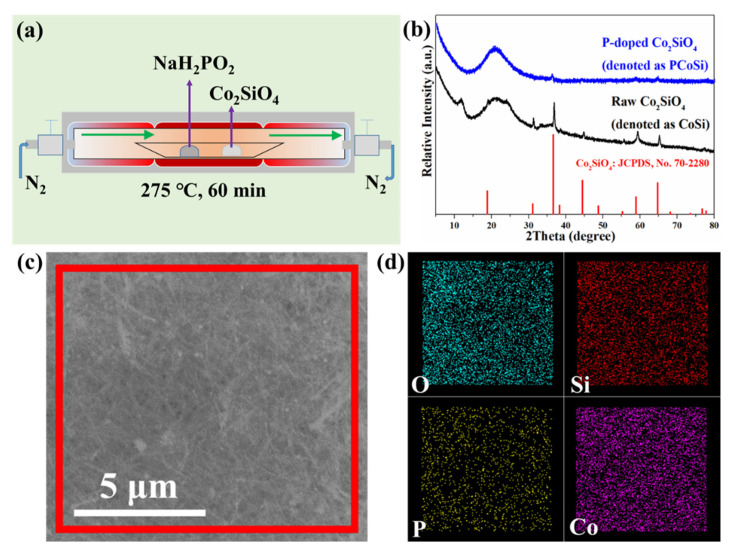
(**a**) Schematical description of the synthetic route of PCoSi; (**b**) XRD patterns of CoSi and PCoSi; (**c**) A SEM image of PCoSi and (**d**) its corresponding elemental mapping images obtained from the red square in (**c**).

Figure 1a shows the synthetic route of PCoSi. As shown in Figure 1a, the doping phosphorus (P) of CoSi is completed by the calcining method. Figure 1b displays the X-ray powder diffraction (XRD) pattern of the as-obtained PCoSi and CoSi. The low crystallinity indicates its main amorphous phase, which is different from the CoSi [35]. CoSi shows some crystallization (Figure 1b). The diffraction peaks of CoSi and PCoSi in Figure 1b are assigned to CoSi (No. 70-2280). The P dopant changes the structure of the initial CoSi to some extent. Figure 1c,d respectively represents a scanning electron microscopy (SEM) image and its corresponding elemental image mappings, which reveal that O, Si, P and Co elements are observed and that they are uniformly distributed.

**Figure 2:**
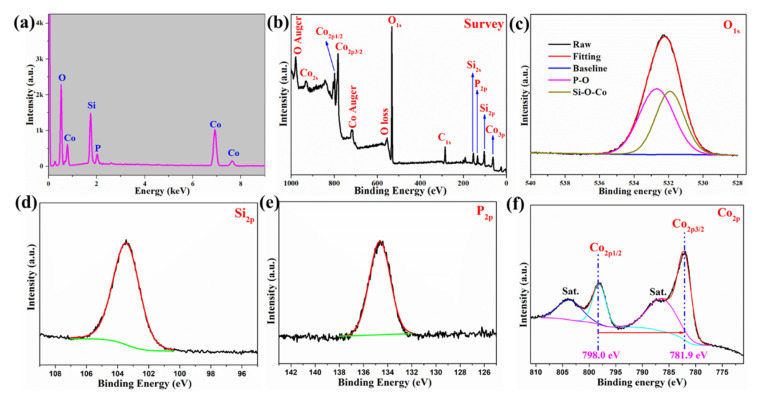
(**a**) The EDS spectrum of PCoSi; (**b**–**f**) XPS spectra of PCoSi: (**b**) full-level spectrum; (**c**) O1s core-level spectrum; (**d**) Si2p core-level spectrum; (**e**) P2p core-level spectrum; (**f**) Co2p core-level spectrum.

Figure 2a depicts the energy-dispersive X-ray spectrometer (EDS) spectrum of PCoSi. Four elements, including O, Si, P and Co, are detected, which agrees with the elemental image mappings (Figure 1d) and the survey X-ray photoelectron spectroscopy (XPS) observation (Figure 2b). The atomic percentage of P in the PCoSi is about 4.82%. Figure 2c–f depicts the core-lever spectra of these elements. The O1s core-level spectrum (Figure 2c) can be split into two peaks at 531.9 and 532.7 eV, corresponding to Si−O−Co and O−P [27]. The Si2p core-level spectrum (Figure 2d) reveals that its binding energy is located at 103.4 eV. The P2p core-level spectrum (Figure 2e) presents a peak located at 134.6 eV, which corresponds to the O−P bond [27]. The Co2p core-level spectrum (Figure 2f) can be separated into four peaks. The binding energies at about 798.0 and 781.9 eV are indexed to Co2p_1/2_ and Co2p_3/2_, respectively. The peaks at about 803.9 and 787.4 eV correspond to their two satellite peaks [26]. The separation of the binding energies between Co2p_1/2_ and Co2p_3/2_ is 16.1 eV, proving that the Co element in the as-obtained PCoSi is in the +2 oxidation state [40]. These results demonstrate that the P is incorporated into CoSi.

Fourier transform infrared spectroscopy (FTIR) and Raman spectra are further provided to characterize the structure of PCoSi. Figure S1a (Supplementary Materials) shows the FTIR spectra of CoSi and PCoSi. The peaks at about 1025, 668 and 460 cm^−1^ are assigned to Si-O-Si, Co-O and Si-O stretching vibrations [36], respectively. A new peak at about 578 cm^−1^ is observed, which is attributed to a P-O stretching vibration [27]. The above results provide an indirect proof for the formation of PCoSi. Figure S1b depicts the Raman spectra of CoSi and PCoSi. No obvious peaks are observed.

**Figure 3:**
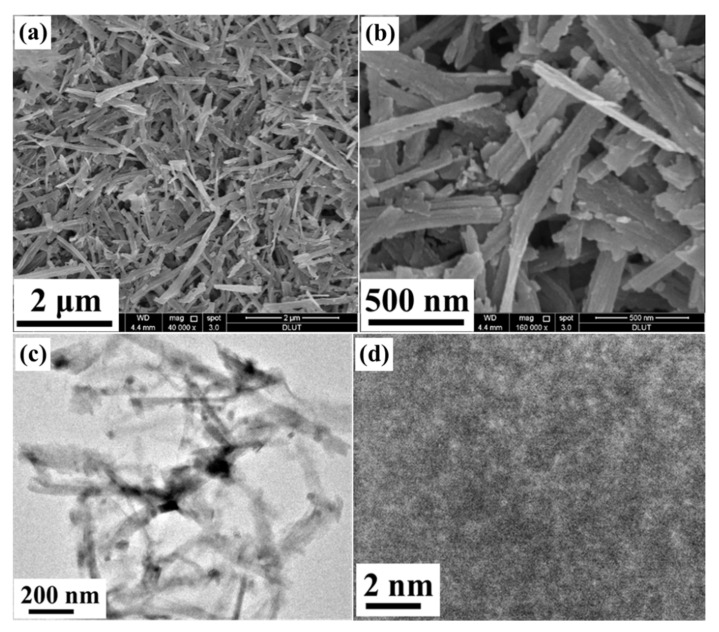
(**a**,**b**) FE-SEM images of PCoSi; (**c**) TEM image of PCoSi; (**d**) HRTEM image of PCoSi.

Figure 3a,b shows the field emission scanning electron microscopy (FE-SEM) images of the as-prepared PCoSi, and a 1D belt-like morphology with a width of ≈100 nm is obtained, in line with the morphology of CoSi (Figure S2a). Besides, the PCoSi belts cross, but no significant sintering occurs, which indicates that the heated treatment with NaH_2_PO_2_ does not destroy the belt-like morphology or lead to agglomeration. Consequently, the pore structures are preserved, which is beneficial for energy storage [41]. Figure 3c depicts its transmission electron microscopy (TEM) image, and the belt-like structure is also seen, supporting the observation of FE-SEM images. These results suggest that the P-doped process does not change the morphology of CoSi (Figure S2b). However, this process changes its structure, which is supported by the HRTEM image (Figure 3d). No clear lattice fringe is observed in HRTEM, proving its amorphous phase. The amorphous structure of PCoSi suggests that the P-doped process reduces the crystallinity, in agreement with the XRD results (Figure 1b). All the above analyses point to the successful fabrication of P-doped CoSi.

**Figure 4:**
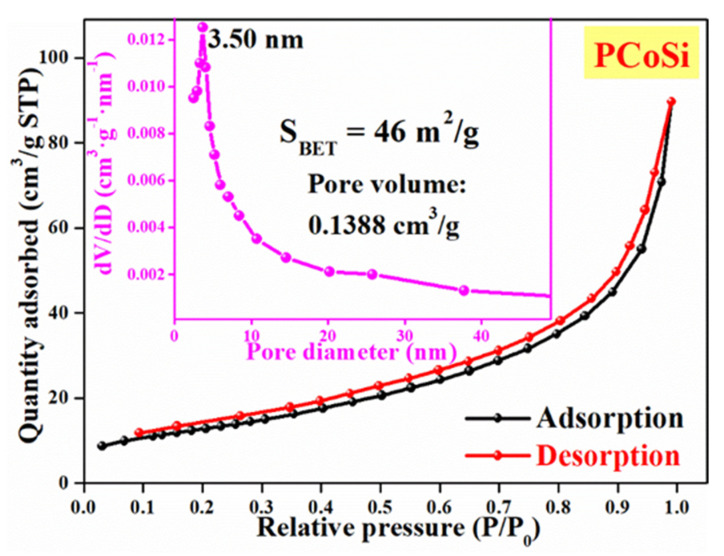
N_2_ adsorption-desorption isotherms of PCoSi, inserting the corresponding pore size-distribution curve.

Figure S3 and Figure 4 display the N_2_ adsorption/desorption isotherms of CoSi and PCoSi, respectively. The as-obtained isotherms are assigned to the Type-IV isotherms according to IUPAC, indicating the mesoporous structures of CoSi and PCoSi. The Brunauer Emmett Teller (BET) specific surface area of PCoSi is 46 m^2^∙g^−1^. The pore volume of PCoSi is up to 0.1388 cm^3^∙g^−1^, and the most probable distribution centers at about 3.5 nm. On the other hand, the BET specific surface area and pore volume of PCoSi (Figure S3) are 54 m^2^∙g^−1^ and 0.2016 cm^3^∙g^−1^, respectively. The porous features of PCoSi are smaller than those of CoSi, which indirectly proves the successful achievement of P dopant. The results suggest that PCoSi is a mesoporous material with a relatively high specific surface area, which can provide numerous active sites for contact between the electrode materials and the electrolyte in order to enhance the redox reactions for charge storage [41]. Furthermore, the P dopant can introduce extra reactive sites to CoSi and enhance its electrochemical properties [27].

**Figure 5:**
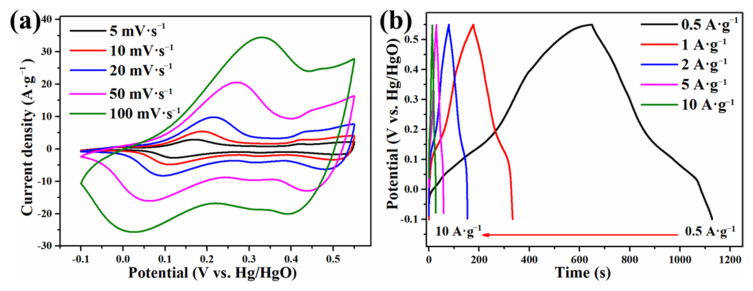
(**a**) CV curves of PCoSi; (**b**) GCD curves of PCoSi.

To show the merits of the as-prepared PCoSi, galvanostatic charge-discharge (GCD), cyclic voltammetry (CV) and electrochemical impedance spectroscopy (EIS) tests were used to estimate its electrochemical properties. The CV curves of PCoSi at various potential intervals reveal that the proper potential window is chosen to be −0.1–0.55 V. Figure S4a and Figure 5a depict the CV curves of CoSi and PCoSi at various scan rates. A similar shape is observed in these curves [35], indicating that the P dopant cannot change the storage mechanism of CoSi. With the increase of the scan rate, the response of the redox peaks also increases, implying a good rate performance [35]. The obvious peaks suggest that the charge storage mechanism originates from the redox [33]. The obvious redox peaks demonstrate a battery-type behavior, and the charge storage is depicted in the following equation:

(1) $${\mathrm{Co}}_{2}^{\mathrm{II}}{\mathrm{SiO}}_{4}+2{\mathrm{OH}}^{-}↔{\mathrm{Co}}_{2}^{\mathrm{III}}{\mathrm{SiO}}_{4}{\left(\mathrm{OH}\right)}_{2}+2e^{-}$$

The doped P may also enhance the electron transfer, and details on P for charge storage should be further studied. GCD curves of CoSi and PCoSi at different current densities are displayed in Figure S4b and Figure 5b. The positions of the platforms in these GCD curves are consistent with the peaks of the oxidation in the CV curves (Figure S4a and Figure 5a). These evidences also support the battery-type behavior of CoSi and PCoSi [42].

**Figure 6:**
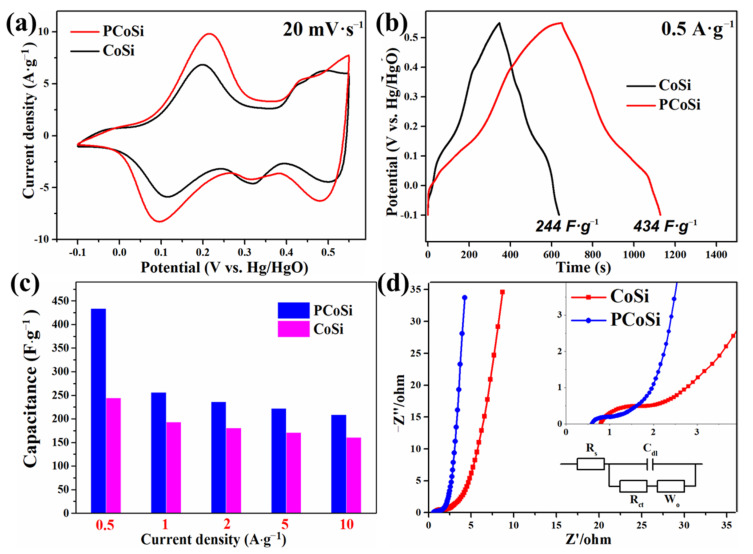
The compared electrochemical properties of CoSi and PCoSi: (**a**) The compared CV curves; (**b**) The compared GCD curves; (**c**) The comparison of the specific capacitance vs. current density of CoSi and PCoSi; (**d**) The comparison of the Nyquist plots of CoSi and PCoSi, inserting their correspondingly enlarged images (top right) and the equivalent circuit diagram (down right).

Figure 6 compares the electrochemical properties of CoSi and PCoSi, demonstrating that the doping of phosphorus can improve the electrochemical performance of CoSi. Figure 6a compares the CV curves between CoSi and PCoSi at 20 mV∙s^−1^. The integral area of PCoSi is larger than that of CoSi, suggesting the higher specific capacitance of PCoSi. The compared GCD curves between PCoSi and CoSi at 0.5 A∙g^−1^ are depicted in Figure 6b, which clearly reveals that the PCoSi displays a larger specific capacitance than that of CoSi because of the longer charge/discharge time of PCoSi. This result agrees well with the observations of the CV curves. The relationship between the specific capacitances and current densities of PCoSi and CoSi is represented in Figure 6c. These values are obtained from the GCD curves in Figure S4b and Figure 5b. The specific capacitances of PCoSi at each current density are much higher than the values of CoSi. Specifically, at 0.5, 1, 2, 5 and 10 A·g^−1^, the specific capacitances of PCoSi (Figure 6c) are 434, 256, 236, 222 and 209 F·g^−1^, while the specific capacitances of CoSi are 244, 193, 181, 171 and 161 F·g^−1^. The exceeded capacitances after the P dopant are 190, 63, 55, 51 and 48 F·g^−1^ at 0.5, 1, 2, 5 and 10 A·g^−1^, respectively.

Figure 6d depicts the EIS spectra of PCoSi and CoSi, which provide the reason for why the electrochemical performance of PCoSi is improved. These EIS spectra consist of two parts: (1) the semicircle of the high frequency region controlled by charge-transfer (the charge transfer resistance, R_ct_, which can be read out by the diameter of the fitting semicircle); (2) the diagonal line of the low frequency region controlled by mass-transfer (the Warburg diffusion resistance, Z_w_, which can be obtained by the slope of the curve). In the high-frequency region, the intercept with the X-axis and the diameter of the semicircle of PCoSi (≈0.6 Ω and ≈0.5 Ω) are smaller than those of CoSi (≈0.8 Ω and ≈1.0 Ω), meaning that there is a slight decrease in the resistances after the P dopant. Meanwhile, in the low-frequency region, its slope is much steeper than that of CoSi, indicating that the Z_w_ of PCoSi is reduced to a much smaller amount than CoSi. These results demonstrate the small resistances (including equivalent series, charge transfer and electrochemical reaction resistances) of PCoSi [43]. Thus, the PCoSi electrode shows an improved electrical conductivity, which enhances the electrochemical performance.

**Figure 7:**
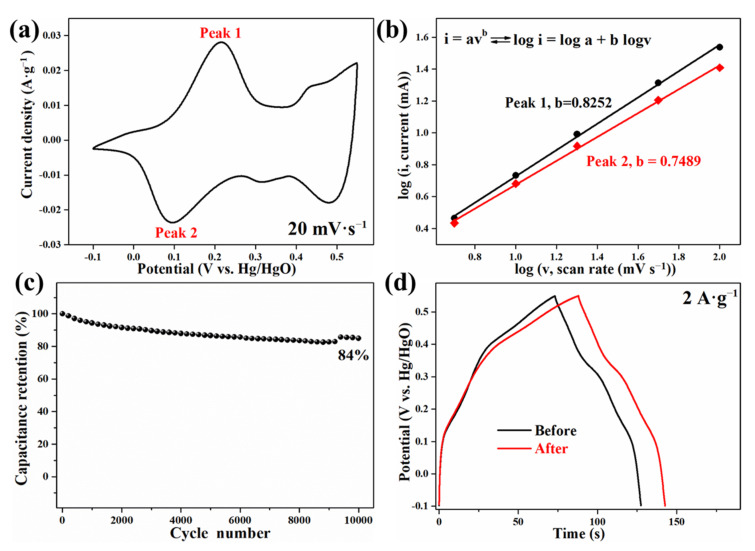
(**a**) The CV curve shows Peak 1 and Peak 2 to illustrate (**b**). (**b**) The plots of log (i) vs. log (v) for the redox current peaks; (**c**) Cycle performance; (**d**) GCD curves of CoSi before and after the calcination at 275 °C for 60 min.

The electrochemical kinetics of PCoSi are provided by Figure 7a,b. It is analyzed by evaluating the correlation between the scanning rate (***v***) and peak current (***i***) based on the CV curves (Figure 5a). Figure 7a gives an example of the peak current. The equation is given as:

***i* = *av*^*b*^** (2)

where ***a*** and ***b*** are coefficients. The ***i*** and ***v*** mean the current (A) and scan rate (mV·s^−1^), respectively. To analyze the dynamic behavior, the ***b*** value is the key parameter. If ***b*** = 0.5, the reaction is mainly a diffusion-controlled behavior. When ***b*** = 1, the reaction is mainly a surface-controlled capacitive behavior. As depicted in Figure 7b, the b value is obtained from the slope of the linear plots. The value is 0.82 in the cathodic process and 0.75 in the anodic process. These results mean that the diffusion-controlled and surface-controlled capacitive (main) behaviors coexist in the charging/discharging process of CoSi electrodes [44].

Figure 7c displays the cycle stability of PCoSi, and the capacitance retention is about 84% after 10,000 cycles. This value proves its superior stability to CoSi (Table S1) [35]. Furthermore, the calcination process has little influence on the electrochemical performances of CoSi, as shown in Figure 7d.

In addition, to investigate the influence of the quantity of P dopant on the electrochemical properties of PCoSi, we carried out comparative experiments. The quantity of initial NaH_2_PO_2_ was fixed to 125 mg, 250 mg and 375 m, respectively. These samples were named PCoSi-1, PCoSi-2 and PCoSi-3. The sample PCoSi-2 (PCoSi represents PCoSi-2 in this work) in the recipe showed the highest specific capacitances (Figure S5).

All the results testify to the fact that the electrochemical properties of CoSi are tremendously enhanced by the P dopant. The achieved electrochemical properties of PCoSi are comparable to or even higher than the state-of-the-art Co-based silicates in Table 1. The excellent electrochemical performances of PCoSi are mainly due to the merits of the 1D structure [34] and P dopant [27]: (1) Its 1D structure facilitates the ion diffusion and electron transfer; (2) The strong nucleophilic P will introduce much more reactive sites.

## 3. Conclusions

In summary, a calcining strategy is developed to synthesize P-doped CoSi, and it shows improved electrochemical properties when used as a SC’s electrode. The as-obtained PCoSi delivers a much higher specific capacitance and cycle stability than that of CoSi, succeeding in the aim to improve the electrochemical properties of CoSi through P doping. This work not only proves that P doping can enhance the electrochemical capabilities of CoSi, but it also opens up a new way to dope P to boost the electrochemical properties of TMSs.

## 4. Materials and Methods

### 4.1. Fabrication of Materials

All chemicals (purchased from Sinopharm Chemical Reagent Co., Ltd., Dalian, China) were used directly without pretreatment. Co-based precursor [Co(OH)_0.8_(C_6_H_5_COO)_1.2_(2C_6_H_5_COOH)_0.04_·1.86H_2_O] and CoSi nanobelts were prepared according to the previous work [35]. Figure 1a schematically illustrates the prepared process of PCoSi. In detail, 250 mg NaH_2_PO_2_ and 25 mg synthesized CoSi were sequentially put in a quartz boat in a tube furnace in N_2_ flow. Then, the tube furnace was heated to 275 °C with 5 °C/min for 60 min. After the calcination, it naturally cooled to room temperature in N_2_ flow, and the sample was collected.

### 4.2. Characterizations

The phase of PCoSi was identified by X-ray powder diffraction (XRD) on a Panalytical X’Pert powder diffractometer (PANalytical B.V., Almelo, The Netherlands) at 40 kV and 40 mA with Ni-filtered Cu Kα radiation. The chemical composition of PCoSi was studied by an energy-dispersive X-ray spectrometer (EDS) and elemental mapping attached to a scanning electron microscope (SEM, QUANTA450, FEI, Hillsboro, OR, USA), and X-ray photoelectron spectroscopy (XPS, ESCALAB250Xi, Thermo Fisher Scientific, Waltham, MA, USA). The Fourier transform infrared spectroscopy (FTIR) pattern of the solid samples was measured using the KBr pellet technique (about 1 wt% of the samples and 99 wt% of KBr were mixed homogeneously, and then the mixture was pressed into a pellet) and recorded on a Nicolet 6700 spectrometer from 4000 to 400 cm^−1^ with a resolution of 4 cm^−1^. Raman spectra were obtained using a Thermo Scientific spectrometer, at an exciting wavelength of 532 nm. The morphologies of PCoSi were observed by field emission scanning electron microscopy (FE-SEM, FEI NOVA NanoSEM 450, FEI) and transmission electron microscopy (TEM, FEITecnai F30, FEI). N_2_ adsorption-desorption isotherms were measured by a Micromeritics-Accelerated Surface Area and Porosimetry system (ASAP 2020M+C, Micromeritics Instrument Co., Norcross, GA, USA).

### 4.3. Electrochemical Characterizations

The preparation of the PCoSi-based electrode used the mature route. PCoSi-based electrodes were prepared using a mixture of 80 wt% of PCoSi, 10 wt% of polyvinylidene difluoride (PVDF) and 10 wt% of carbon black. N-methyl-2-pyrrolidone (NMP) was employed as a solvent. The slurries were coated onto Ni foils and heated at 80 °C for 12 h to remove organic solvent. Then, these foils were pressed onto Ni-grids at 10 MPa. 3 mol·L^−1^ KOH aqueous electrolyte was used as electrolyte. The electrochemical properties of the PCoSi electrode were measured using a three-electrode method by galvanostatic charge-discharge (GCD), cyclic voltammetry (CV) and electrochemical impedance spectroscopy (EIS) tests. Electrochemical tests were performed on a CHI-660D electrochemical work station (Chenghua, Shanghai, China). The specific capacitance (***C***) of PCoSi by GCD is calculated from the following equation:

(3) $$C=\frac{I\cdot \Delta t}{m\cdot \Delta V}$$

where ***C*** (F·g^−1^) means the specific capacitance, ***I*** (A) equals the discharge current, Δ***t*** (s) denotes the discharge time, ***m*** (g) represents the mass of PCoSi in the electrode, and Δ***V*** (V) is the potential window.

## Figures and Tables

**Table 1:** Comparison of the specific capacitance of the as-obtained PCoSi with the state-of-the-art silicate materials in the literature.

Co-Based Silicates	Electrolyte	Potential	Capacitance	Cycle	Reference
CoSi/GO	3 M KOH	−0.1–0.55	511 F g^−1^, 0.5 A g^−1^	84%, 10,000 cycles	[35]
CoSi@MnSiO_3_	3 M KOH	−0.1–0.55	309 F·g^−1^, 0.5 A·g^−1^	64%, 10,000 cycles	[34]
CoSi NN/RGO	3 M KOH	−0.1–0.55	483 F·g^−1^, 0.5 A·g^−1^	58%, 10,000 cycles	[30]
CoSi@MnO_2_	3 M KOH	−0.5–0.6	490.4 F·g^−1^, 1.0 A·g^−1^	45%, 5000 cycles	[37]
Co_3_(Si_2_O_5_)_2_(OH)_2_	6 M KOH	0.1–0.55 V	237 F g^−1^, 5.7 mA cm^−2^	95%, 150 cycles	[45]
Co_3_Si_2_O_5_(OH)_4_	6 M KOH	0–0.5 V	570 F g^−1^, 0.7 A g^−1^	—	[32]
CoSi	3 M KOH	0–0.5	453 F·g^−1^, 0.5A·g^−1^	89%, 10,000 cycles	[40]
(Ni, Co)_3_Si_2_O_5_(OH)_4_/C	3 M KOH	−0.8–0.6	226 F·g^−1^, 0.5 A·g^−1^	99%, 10,000 cycles	[26]
CoSi@Ni(OH)_2_	3 M KOH	−0.1–0.55 V	1101 F·g^−1^, 1.0 A·g^−1^	46%, 4000 cycles	[38]
C/Co_3_Si_2_O_5_(OH)_4_	3 M KOH	−0.05–0.4	1600 F g^−1^, 1 A g^−1^	91%, 6000 cycles	[33]
Co_2.18_Ni_0.82_Si_2_O_5_(OH)_4_	3 M KOH	0–0.5	981 F g^−1^, 0.7 A g^−1^	99%, 6000 cycles	[13]
(Ni, Co)_3_Si_2_O_5_(OH)_4_	1 M KOH	0–0.5 V	144 F g^−1^, 1 A g^−1^	99.3%, 10,000 cycles	[31]
CoSi	6 M KOH	0–0.5	214 F·g^−1^,1 A·g^−1^	83%, 10,000 cycles	[12]
e-CoSi	6 M KOH	0–0.5	267 F·g^−1^,1 A·g^−1^	90%, 10,000 cycles	[12]
PCoSi	3 M KOH	−0.1–0.55	437 F·g^−1^, 0.5 A·g^−1^	84%, 10,000 cycles	This work

## Data Availability

No information.

## Supplementary Materials

The following are available online, Figure S1: FTIR spectra and Raman spectra of CoSi and PCoSi. Figure S2: FE-SEM and TEM image of CoSi. Figure S3: N2 adsorption-desorption isotherms of CoSi. Figure S4: Electrochemical properties of CoSi. Figure S5: GCD curves of PCoSi synthesized using different contents of NaH2PO2.

## Sample Availability

Samples of CoSi and PCoSi are available from the authors.
