# Supplementary material for: Fabrication of Phosphorus-Doped Cobalt Silicate with Improved Electrochemical Properties
Source: Molecules. 2021 Oct 15;26(20):6240. doi: 10.3390/molecules26206240 (PMC8539304; doi:10.3390/molecules26206240)
Supplement: Supplementary file 1 [file molecules-26-06240-s001.zip › molecules-1365808-supplementary.pdf]

Supplementary Material

for

Fabrication of phosphorus doped cobalt silicate with improved electrochemical properties

Jie Ji[1], Yunfeng Zhao[2], Yifu Zhang[2], Xueying Dong[2], Changgong Meng[2], Xiaoyang Liu[1,*]

[1] State Key Laboratory of Inorganic Synthesis and Preparative Chemistry, College of Chemistry, Jilin University, Changchun 130012, PR China; jijie1218@mails.jlu.edu.cn (J. Ji)

[2] Affiliation 2 State Key Laboratory of Fine Chemicals, School of Chemical Engineering, Dalian University of Technology, Dalian, 116024, China; yfdlut@163.com (Y. Zhao); yfzhang@dlut.edu.cn (Y. Zhang); dxy1123@mail.dlut.edu.cn (X. Dong); cgmeng@dlut.edu.cn (C. Meng)

* Correspondence: liuxy@jlu.edu.cn

Figure S1

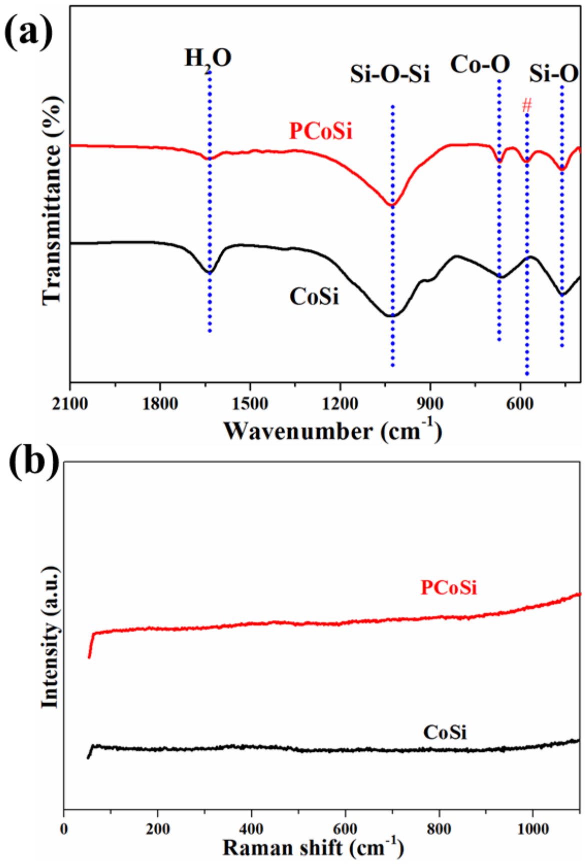

Figure S1. (a) FTIR spectra and (b) Raman spectra of CoSi and PCoSi.

Figure S2

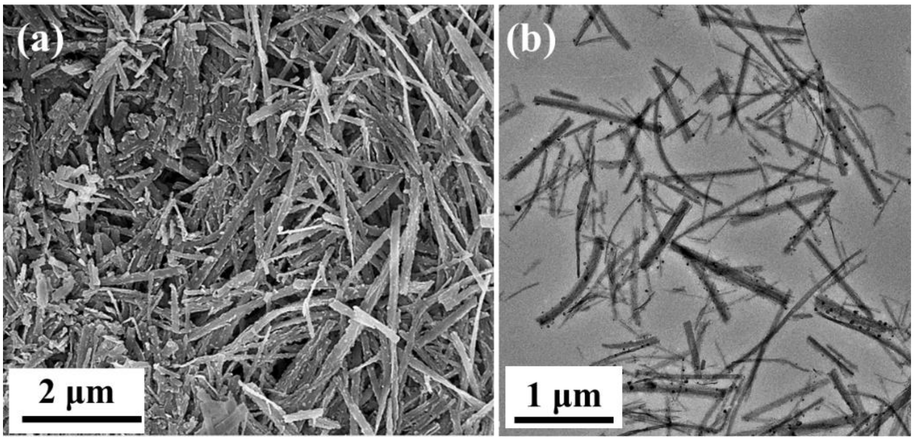

Figure S2. (a) FE-SEM and (b) TEM image of CoSi.

Figure S3

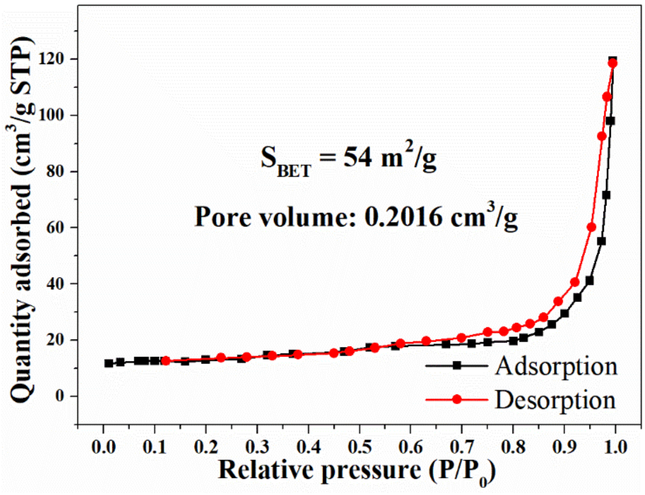

Figure S3. N_2 adsorption-desorption isotherms of CoSi.

Figure S4

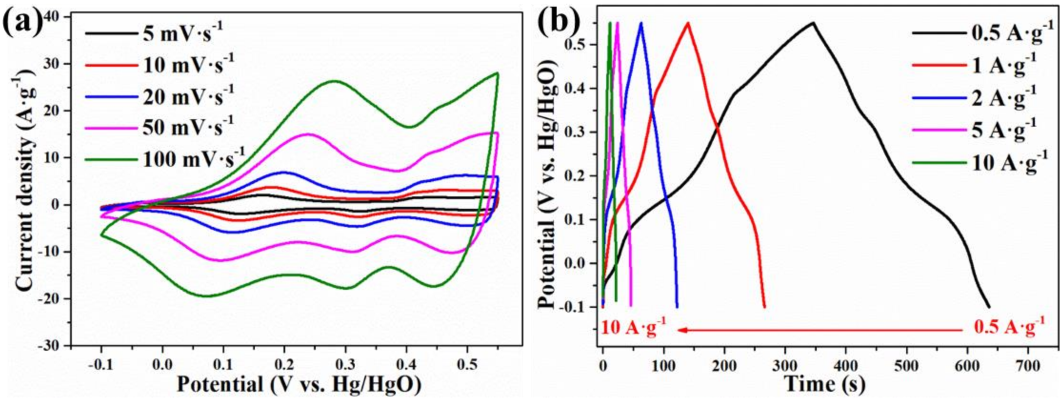

Figure S4. Electrochemical properties of CoSi: (a) CV curves at various scan rates; (b) GCD curves at various current densities.

Figure S5

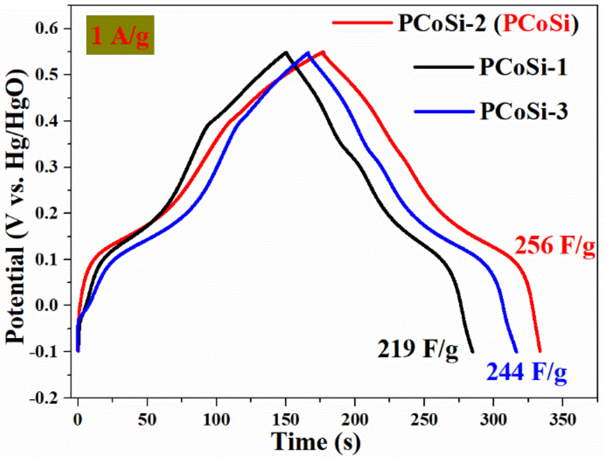

Figure S5. GCD curves of PCoSi synthesized using different contents of NaH_2PO_2.

PCoSi-1: 125 mg NaH_2PO_2

PCoSi-2: 250 mg NaH_2PO_2

PCoSi-3: 275 mg NaH_2PO_2
